# Assessing Vulnerability to Urban Heat: A Study of Disproportionate Heat Exposure and Access to Refuge by Socio-Demographic Status in Portland, Oregon

**DOI:** 10.3390/ijerph15040640

**Published:** 2018-03-30

**Authors:** Jackson Voelkel, Dana Hellman, Ryu Sakuma, Vivek Shandas

**Affiliations:** 1 School of Urban Studies and Planning, Portland State University, Portland, OR 97201, USA; jvoelkel@pdx.edu (J.V.); dhellman@pdx.edu (D.H.); 2 Peace Winds Japan, Tokyo 151-0063, Japan; lyu.sakuma@gmail.com

**Keywords:** urban heat, vulnerability, environmental justice, heat exposure, resilience

## Abstract

Extreme urban heat is a powerful environmental stressor which poses a significant threat to human health and well-being. Exacerbated by the urban heat island phenomenon, heat events are expected to become more intense and frequent as climate change progresses, though we have limited understanding of the impact of such events on vulnerable populations at a neighborhood or census block group level. Focusing on the City of Portland, Oregon, this study aimed to determine which socio-demographic populations experience disproportionate exposure to extreme heat, as well as the level of access to refuge in the form of public cooling centers or residential central air conditioning. During a 2014 heat wave, temperature data were recorded using a vehicle-traverse collection method, then extrapolated to determine average temperature at the census block group level. Socio-demographic factors including income, race, education, age, and English speaking ability were tested using statistical assessments to identify significant relationships with heat exposure and access to refuge from extreme heat. Results indicate that groups with limited adaptive capacity, including those in poverty and non-white populations, are at higher risk for heat exposure, suggesting an emerging concern of environmental justice as it relates to climate change. The paper concludes by emphasizing the importance of cultural sensitivity and inclusion, in combination with effectively distributing cooling centers in areas where the greatest burden befalls vulnerable populations.

## 1. Introduction

Extreme heat poses a growing threat to human populations, with numerous implications for public health, economic stability, and quality of life [1,2,3]. Past heat waves have had devastating, deadly outcomes worldwide [4,5,6], and such events are expected to increase in intensity, frequency, and duration as climate change progresses [7,8]. Although human settlements of any type may experience the negative effects of extreme heat, these are and will continue to be most pronounced in urban areas, the development practices of which are highly correlated with rising temperatures [9,10,11]. Currently, more than 50% of the world’s population is located in urban areas, and that figure is expected to reach over 66% by 2050 [12]; with so many people potentially at risk of exposure, it is imperative that local governments and planning practitioners recognize varying degrees of vulnerability among urban residents.

Urban heat events—defined as those above the 90th percentile of historic temperatures [13]—are an environmental stressor, placing economic, infrastructure, and human health burdens on society [14,15,16]. As a stressor, urban heat can create vulnerabilities, which may be understood as a combination of three factors [17]: exposure, sensitivity, and adaptive capacity. *Exposure* refers to an individual’s contact with a stressor, either from living, working, or spending time in an affected location. *Sensitivity* is the point at which exposure becomes dangerous to an individual’s health [18]. Finally, *adaptive capacity* refers to one’s ability to change exposure or sensitivity, or to cope with an extreme event. Regarding urban heat, indicators believed to enhance adaptive capacity include high income, social cohesion, and knowledge of hazardous environments [19,20]. Given these conditions, it may be reasonable to categorize extreme heat exposure as an environmental justice issue.

The phenomenon central to this study is the urban heat island (UHI) effect, which has been known to researchers since the mid-19th century, and indicates a strong correlation between urban environments and high temperatures [21,22]. Impervious surfaces and anthropogenic activity within cities portend rising temperatures, as does the relative scarcity of heat-ameliorating elements such as trees and grasses [23,24,25]. A higher frequency of regional extreme heat events, as such, will amplify temperatures [8], and generate UHI in areas that have greater amounts of heat absorbing surfaces. While early studies focused on the comparative temperatures between urban and non-urban regions, the emergence of mobile sensors, highly accurate global positioning systems, and computational software allows for the comparison of intra-urban spaces, measuring variation in temperature distribution across a single city [26,27]. Past research has utilized infrared satellite data for this purpose [10,28,29], though vehicle-based traverse measurements (used in this study) can provide a detailed representation of heat exposure at a smaller scale [30,31,32,33].

From an environmental justice point of view, the existing research emphasizes point source pollution [34,35,36], though it has become clear that climate change affects communities differentially and creates novel impacts never before witnessed in traditional environmental research. As such, we argue that climate change is catalyst for injustice. While some of the effects can be easily observed at the national level, particularly in the world’s poorest countries [37,38], there is relatively little understanding of the impact at a more granular scale. Needed are approaches—methodological, conceptual, and pragmatic—that help us to identify those communities disproportionately affected by UHI, and strategies that can help to reduce vulnerabilities. This study provides new evidence of disproportionate exposure to climate change at a local level, as well as access to refuge, an understudied facet of adaptive capacity.

Previous studies indicate a relationship between socio-demographic factors and heat-related morbidity/mortality [39,40]. Income is quite predictive of vulnerability (inverse relationship) [41,42,43], though it is possible that other indicators also play a role. If so, urban heat exposure may be framed as an environmental justice or ‘climate justice’ [44] issue, disproportionately affecting marginalized socio-demographic groups with limited adaptive capacity. This study aims to identify such populations in Portland, Oregon by assessing (1) disproportionate heat *exposure* among socio-demographic groups; and (2) disproportionate access to refuge (either public refuge facilities or residential central air conditioning), resulting in heightened or lowered *adaptive capacity*. An in-depth statistical and spatial analysis will reveal significant, inequitable relationships, validating the application of an environmental justice lens in addressing urban heat resilience.

## 2. Materials and Methods

### 2.1. Study Area

Our assessment occurs in a Pacific Northwest city of the United States. The City of Portland, Oregon is located at approximately 45.5° North, 122.6° West, at the confluence of the Willamette and Columbia Rivers. The city covers approximately 345 square kilometers, with of a population of nearly 640,000 as of 2016 [45]. The City of Maywood Park, Oregon is located within northeast Portland and, though an enclave of the City of Portland, has been excluded from the study. Due to the fact that summer temperatures have an average monthly highs of 22.7 °C, 26.4 °C, and 26.7 °C for June, July, and August, respectively [46], Portland offers several advantages to conducting an assessment of disproportionate effects of urban heat. First, historical high temperatures have rarely exceeded 35 °C, which reduces public concern for heat related illnesses. Second, as of 2013, fewer than 35% of households had air conditioning [47], and communities may not have immediately available private residences to consider refuge from heat waves. Finally, Portland Climate Action Plan (2015) explicitly addresses the importance of reducing disproportionate exposure to urban heat waves, yet few actions have materialized.

### 2.2. Data

Three main data types were used in this study, all at the U.S. census block group level: socio-demographic indicators, distribution of ambient temperatures in the study location, and refuge availability. Socio-demographic data used include: income (percent of the population below 50% of the poverty line); race (percent of the population who do not self-identify as white); education (percent of the population over 25 years old without a high school degree or equivalent); age (percent of the population over 65 years old that lives alone); and English speaking ability (percent of the population that claims to have poor or no English skills). Obtained from the U.S. Census Bureau’s American Community Survey 5-year estimates, 2009–2013 [47], these data reflect categories into which individuals have self-identified. Another, non-census piece of data used to highlight socio-demographic status is presence of affordable housing, obtained from Oregon Metro’s Regional Land Information System [47]. In order to differentiate “low” and “high” categories used in the analysis, a model-based clustering algorithm was used to split each variable [48].

Following an established protocol [13], we collected approximately 60,000 temperature readings during one day of an extreme heat event on 25 August 2014, in Portland, Oregon, when the average temperature during the hottest hour of the day was in the 90th percentile of 30-year historic daily temperatures for the study region. We sampled temperatures for one hour at 3 times during the day (6 a.m., 3 p.m., and 7 p.m.) using vehicle traverses (cars with a mounted temperature sensor and global positioning system (GPS)) in six predetermined sections of the city. The temperature sensor consisted of a type T-fine (30 gauge) thermocouple in a plastic shade tube (12 cm in length and 2.5 cm in diameter) mounted on the passenger-side window approximately 25 cm above the roof of each of 5 vehicles deployed. Each temperature sensor was connected to a data-logging device with an estimated system accuracy of ±0.5 °C and a 90% response time of less than 60 s in 1 m/s airflow. A GPS unit on each vehicle paired temperature measurement and location.

Based on the results from the temperature collection and subsequent modeling, we created three separate heat surfaces, which are continuous descriptions of temperature variation across the study region, corresponding to the three time periods. The resulting maps consisted of a 32-bit floating point 1-meter raster format and contain 449,359,188 pixels for each of the three time periods [13,49]. These three urban heat models were created using random forest machine learning on temperature data collected using vehicle-based traverse measurements. Multiple land uses are included in the model (e.g., tree cover, building volume), and the temperatures derived are representative of the underlying urban form. Earlier research suggests that evening temperatures can have the greatest impact on human health, in part due to the exposure overnight, when physiological responses [50,51,52] can be acute among those with pre-existing health conditions. As a result, this study utilizes the evening temperature model in an attempt to identify areas with prolonged exposure to high temperatures. The 7 pm model has an R^2^ of 0.9715 and an RMSE of 0.2078. Using “zonal statistics” in ArcGIS (ESRI, Redlands, CA, USA), average UHI temperature was calculated for each census block, ranging from 26.4 to 30.1 °C. This aggregation method simplifies the UHI dataset, however this alteration of the raw data is deemed worthwhile in order to assess relationships with demographic data. Additionally, this is a common practice in geographic analysis [15,53,54].

In the context of this study, “refuge” refers either to public cooling facilities, or availability of central air conditioning in one’s home; in other words, the availability of coping mechanisms. Public heat refuge data were obtained from the Multnomah County Office of Aging, Disability and Veterans Services [55]. These include three County cooling centers; 33 places to play in the water; 59 libraries; and 73 community centers. The heat refuges were geocoded using Google Earth. Nine out of 33 places to play in the water are not free for personal use, but are treated as such for the purpose of this study. Residential Central Air Conditioning (CAC) data were obtained from the Multnomah County Assessment Office [55].

### 2.3. Analysis

This study assessed multiple facets of vulnerability through the use of mixed spatial and statistical methods, with the aim of identifying not only those hottest areas of the city, but also trends of socio-demographic disparity. Elements considered include exposure of sensitive populations, as well as their ability to cope with heat by accessing refuge.

First, heat exposure was determined by mapping UHI data at the census block group (CBG) level. Using the “raster” package in R statistical software, the mean of all pixels falling geographically within an individual CBG polygon was appended onto that polygon’s data table, resulting in a visual representation of spatial temperature distribution. This indicated areas of the city most exposed to extreme heat.

Second, the relationship between various socio-demographic groups and high-exposure areas was assessed using the Student’s *t*-test method, where α = 0.05 for all tests. This method reveals which sensitive groups, if any, are disproportionately exposed to extreme heat conditions. In each case, two groups are compared: those with low adaptive capacity characteristics, and those with high adaptive capacity characteristics. Indicators included in this analysis, bifurcations of each, and hypotheses tested are as follows.

H0: Average of low income population − Average of high income population = 0

H1: Average of low income population − Average of high income population > 0

where:

Average of low income population = Mean temperature of low income block group

Average of high income population = Mean temperature of high income block group

  

H0: Average of non-white population − Average of white population = 0

H1: Average of non-white population − Average of white population > 0

where:

Average of non-white population = *Mean temperature of block groups with **large** non-white population*

Average of non-white population = *Mean temperature of block groups with **small** non-white population*

  

H0: Average of low education population − Average of high education population = 0

H1: Average of low education population − Average of high education population > 0

where:

Average of low education population = *Mean temperature of block groups with **large** population with less education*

Average of low education population = *Mean temperature of block groups with **small** population with less education*

  

H0: Average of isolated elderly population − Average of accompanied elderly population = 0

H1: Average of isolated elderly population − Average of accompanied elderly population > 0

where:

Average of isolated elderly population = *Mean temperature of block groups with **large** population of isolated elderly*

Average of isolated elderly population = *Mean temperature of block groups with **small** population of isolated elderly*

  

H0: Average of low English proficiency population − Average of English proficiency = 0

H1: Average of low English proficiency population − Average of high English proficiency population > 0

where:

Average of low English proficiency population = *Mean temperature of block groups with **large** population* low English proficiency

Average of low English proficiency population = *Mean temperature of block groups with **small** population with* low English proficiency

  

H0: Average of population in affordable housing − Average of population in non-affordable housing = 0

H1: Average of population in affordable housing − Average of population in non-affordable housing > 0

where:

Average of population in affordable housing = *Mean temperature within 100 m of affordable housing*

Average of population in non-affordable housing = *Mean temperature within 100 m of non-affordable housing*

Third, this study examined the accessibility of refuge for various populations, broken out into specific racial categories, as well as elderly (over 65 years) and young children (under 5 years) age groups. The race groups included in the analysis are white; black or African American; American Indian or Alaskan Native (AIAN); Asian; Native Hawaiian and other Pacific Islander (NHPI); and Hispanic or Latino. Access to public heat refuges was calculated for walking speeds of slow, normal, and fast. Maximum acceptable walking time was set at 15 min, and analyzed based on average walking speeds for sedentary elderly (1.4 km/h), average elderly (3.5 km/h), and active adults (5.6 km/h) [56]. These distances (0.35, 0.875, and 1.4 km, respectively) were applied using “network distance analysis” in ArcGIS to establish heat refuge catchment areas.

Additionally, differences in walking access to refuges, temperature exposure, and access to residential central air conditioning (CAC) were assessed for the aforementioned groups using covariance analysis. Using GeoDa’s “scatter plot” function, percentages of residents with specific characteristics were used as X variables, and the accessibility of heat refuges, UHI, and the prevalence of CAC were used as Y variables (Table 1).

## 3. Results

Results have been divided into three sections. We begin by providing background on the UHI and its integration with the CBG data. We follow with outputs from statistical analyses, which identify relationships between heat exposure and specific socio-demographic groups. Finally, we identify the accessibility of heat refuge options (public cooling centers or central air conditioning) to those who have a low level of adaptive capacity.

### 3.1. Ambient Temperature Distribution

**Figure 1:**
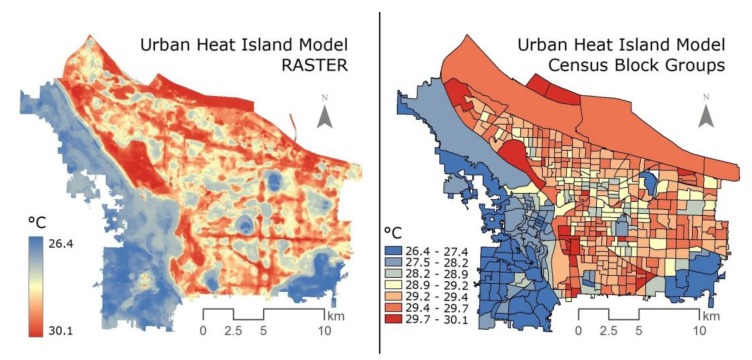
Comparison of the original raster format of the distribution of ambient urban heat (**left**) and the transformed block group-based urban heat dataset (**right**).

The UHI model employed shows a concentration of high-heat areas to the east side of the city, while the west side of the city is relatively cool (Figure 1). Also, we note two implications of converting heat data to CBG. First, the block groups are not coterminous with the UHI data; the boundaries do not exactly overlap, which means that each block group draws from the nearest temperature. Second, since the UHI map is at 1 m resolution and the block groups are much larger, all temperature values within a CBG were averaged. Although these limitations may reduce the overall accuracy of the precise temperature in each CBG, our purpose is to evaluate broad relationships between socio-demographics and UHI, rather than a precise household-scale assessment.

### 3.2. Heat Exposure by Socio-Demographic Group

The results of statistical *t*-tests (Table 2) reveal significant relationships between heat exposure and populations that are low-income, non-white, minimally-educated, or poor English speakers; all of these socio-demographic groups, as well as those living in affordable housing, experience higher temperatures than their wealthy, white, educated, English-speaking counterparts. Isolated elderly is the only tested indicator that did not significantly correlate with higher temperatures.

### 3.3. Exposure and Access to Refuge

**Figure 2:**
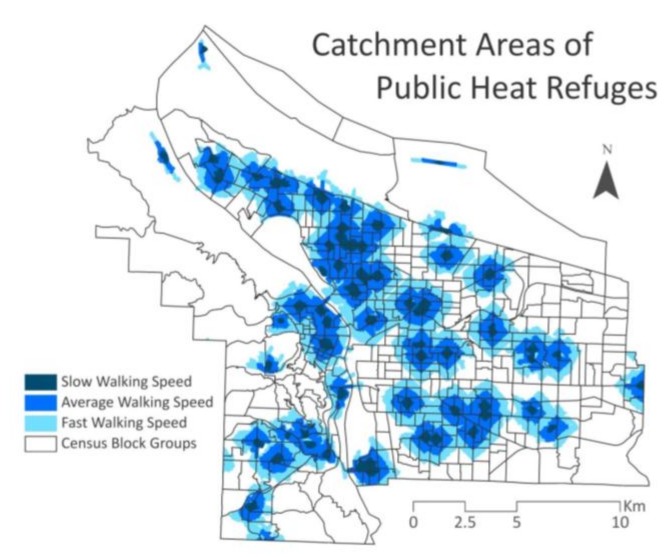
Catchment areas of public heat refuge access for slow, average, and fast speeds.

The network distance analysis of public refuge access shows that 3.4–32.7% of the city’s population can access a refuge on foot, depending upon walking speed (Table 3, Figure 2). These cooling centers are most numerous in North and Northeast Portland, while the farthest eastern and western regions of the city offer fewer options for public refuge.

Based on the results of covariance analyses, distinct inconsistencies emerge and define disproportionate exposure to high temperatures, and accessibility to CAC and public refuges (Table 4). The figures in this table represent expected changes in the dependent variable for a single-unit increase of the independent variable in question. For example, the −1.515 value between “White” and “UHI” indicates that for a 100% increase in white population, a 1.515 degree Celsius decrease in temperature would be expected.

#### 3.3.1. Exposure to Urban Heat

The white and elderly populations have a negative relationship with UHI (−1.515 and −2.114, respectively), which is statistically significant. This means that census blocks with a higher number of white residents and older adults as a percentage of the total census block population are more likely to have lower temperatures during an urban heat event. For example, for every 10% increase in the white population, temperatures are lower by 0.1515 °C on average during a heat event. By contrast, a larger black/African American population, along with NHPI, Hispanics, and young children all have a positive linear relationship with UHI. For example, the coefficient for NHPI is 3.471, meaning that for every 10% increase in NHPI, of the total census block population, temperatures are higher by 0.3471 °C on average. The share of AIAN and Asians do not have a statistically significant relationship. This analysis is based solely on demographic characteristics as they relate to temperature and does not explicitly account for the presence of buildings, trees, or other factors which influence urban heat. However, it may be inferred that those groups experiencing the highest temperatures are located in areas which lack heat-ameliorating infrastructure, or possess built urban features that exacerbate heat [21,22].

#### 3.3.2. Central Air Conditioning (CAC) Units per Area

Only the white population has a significant positive relationship with CAC per area (km^2^). On the other hand, only young children have a negative linear relationship with CAC, which is statistically significant. For every 10% increase in white population, of the total census block population, CAC units are likely to be higher by 9.6 units per square km, and for every 10% increase in young children, CAC units are likely to be less by 30.5 units on average. The share of black/African American residents, AIAN, Asians, NHPI, Hispanics, and the elderly do not have a statistically significant coefficient. 

#### 3.3.3. Public Cooling Centers

When assuming the average walking speed, only the black/African American population has a positive relationship with accessibility to public heat refuges. On the other hand, Asians and the elderly have a negative linear relationship with public heat refuges in the city. For every 10% increase in black/African American population of the total census block population, the residents have more access by 0.22 public heat refuges on average, and for every 10% increase in Asians, the residents have less access by 0.20 public heat refuges. Other tested socio-demographic characteristics do not have statistically significant relationships with the accessibility to public heat refuges.

## 4. Discussion

This study examined socio-demographic factors in relation to the distribution of urban heat in an attempt to better understand vulnerability based on (1) disproportionate heat *exposure* among socio-demographic groups; and (2) disproportionate access to refuge (either public cooling facilities or residential central air conditioning), resulting in heightened or lowered *adaptive capacity.* Overall, results indicate that populations with low adaptive capacity characteristics also experience high exposure, and that access to refuge is significantly influenced by socio-demographic status.

A series of Student’s *t*-tests were performed to test the hypotheses that the difference between “high” and “low” adaptive capacity groups were significantly greater than 0. The results of the study, with the exception of the variable isolated elderly, allow rejection of the null hypothesis, and indicate that populations with characteristics of low adaptive capacity do experience higher temperatures than those with high adaptive capacity within the study area. Additionally, the analysis showed significantly higher temperatures in the area directly surrounding affordable housing when compared to a random sample of non-affordable (i.e., regular) housing from similar block groups. 

We focused on isolated elderly specifically because they have historically been disproportionately impacted by heat waves in other parts of the U.S. [39,40], though similar patterns are not statistically significant in the City of Portland. In fact, the observed non-significance of the isolated elderly (percent of the population 65+ years old and who live alone) could be related to the spatial nature of the census block group geographies. A test for spatial autocorrelation conducted using Moran’s I [56] showed that, while census block groups with a high percent of isolated elderly have statistically significant clustering (z-score = 2.921, where 0 is random; *p*-value = 0.0035), they are far more random in spatial distribution than the variables for extreme poverty (z-score = 6.411; *p*-value ≈ 0), high racial diversity (z-score = 15.475; *p*-value ≈ 0), poor English skills (z-score = 15.673; *p*-value ≈ 0), and low education (z-score = 17.787; *p*-value ≈ 0). This notable difference in spatial autocorrelation shows that block groups with high levels of isolated elderly populations are more randomly distributed than the other socio-demographic variables, thus increasing the chances that they will have a more randomized exposure to extreme heat and a less significant Student’s *t*-test result.

The accessibility analysis revealed that walking speeds, as they relate to the distribution of cooling centers, greatly affect the percentage of areas in the city having access to heat refuge. At the slower walking speed (1.4 km per hour), only 3.4% of residents have access; at the average speed (3.5 km per hour), the percentage increases to 16.9%; and at the fast speed (5.6 km per hour), it increases to 32.7%. This finding reveals that even in the best case scenario (fast speed), less than one third of the population can access a public heat refuge. This may be especially meaningful for individuals with mobility challenges, such as those using wheelchairs, those with pre-existing health conditions, and bedridden patients, though such groups have not been included in this study.

The covariance analysis found racial and age-related disparities in distribution of UHI, CAC, and walkable access to heat refuges. Risk factors concentrate on some socio-demographic groups, especially young children. They are more likely to live in census blocks which are hotter during urban heat events, and with a smaller number of CAC units. In contrast, white populations tend to live in census blocks with less UHI effect, and more CAC units. Black/African American populations tend to have better accessibility to public heat refuges, which may prove helpful if they are concentrated in high-heat census block groups. While analyses focusing on environmental justice have found that non-white communities are disproportionately living near point sources, urban heat and the access to refuge arguably represent novel concern that may further deepen the inequities in society.

This study does not offer a complete exploration of the factors which determine why certain socio-demographic groups cluster in areas experiencing higher temperatures. This is a complex question that would require a complete study of its own, though some of the likely contributing factors are known to researchers. Urban development patterns often feature lower rents in areas near large roads and buildings [57], both of which can amplify urban heat effects. Assuming individuals with limited financial means seek out lower rent, this increases their likelihood of locating in areas with higher heat stress. A second possible factor relates to socialization and, in some cases, spatial isolation of minority communities. Such groups have a history of building social capital by co-locating in neighborhoods, as well as being coercively isolated in specific locations [58,59,60]; this could result in apparently heightened heat exposure for such groups, simply due to their proximity. In the case of Portland, local development practices have typically placed large trees and other heat-ameliorating features in higher-income neighborhoods [61], exacerbating heat exposure of low-income and minority communities who have historically been excluded from these areas. These are multifaceted relationships that differ across cities, and are outside the purview of this study. However, it is useful to consider the underlying causes of physical clustering and resulting exposure.

One major drawback to the analyses in this study is the geographic format of the data. The irregular polygon geometry of the census block group data relies on areal aggregation to protect the anonymity of individuals; this aggregation of population and heat data into enumeration units can ‘smooth’ the dataset, eliminating extreme highs and lows in the process of representing the data with a single mean value. This complication is difficult to avoid, as the block group geometries employed in this study are the highest resolution datasets available with the required socio-demographic information. Additionally, the Modifiable Areal Unit Problem (MAUP) may introduce error when using enumeration units such as census block groups [62]. A potential alternative to this census-based study would be to create an entirely new survey of randomly sampled households in the region. This potential new study could allow for a building-level analysis similar the one performed here for affordable housing, but for all socio-demographic variables. A survey with a sample size high enough for statistically sound inference and analysis would be time consuming and costly, however it could potentially reveal more accurate or meaningful results.

This study is also lacking in a key piece of information which would provide a more complete understanding of vulnerability; though exposure and adaptive capacity have been well explored, sensitivity has not, mainly because reliable data on health, genetics, and lifestyle choices are difficult to obtain. For this reason, the definition of vulnerable populations may not be fully accurate because we do not accurately know whether individuals do not, in fact, have access to other forms of refuge (e.g., ductless heat pump, swimming pool, alternative residences, etc.). At the same time, at the population level, the present study finds significant associations between high exposure and low adaptive capacity, which provide meaningful direction for decision makers to prioritize those areas and groups that are likely to be at high risk.

### Next Steps for Practitioners

The results of this study may serve as a guide for practitioners in Portland, Oregon, directing attention to those areas of the city most at risk of extreme heat exposure. However, socio-demographic indicators can only reveal general characteristics of a population; as such, community engagement in these priority areas will be a key strategy moving forward. These results suggest that practitioners will need to meet with community members directly to better understand what they experience during a heat wave, how they adapt, and what they perceive their needs and strengths to be. Rather than offering strictly external monetary or technological support, sustainable solutions may be reached by working with local organizations and individuals to build internal capacity.

Given the diverse nature of marginalized groups exposed to extreme heat, it will be helpful for the City of Portland and Multnomah County to release heat-related materials for such an audience. Information regarding public refuges and heat safety, as well as heat wave warnings should be issued in multiple languages and formats (print, online). Messaging tailored to specific groups may also be helpful. It is further recommended that government agencies work with community organizations to disseminate information and provide refuge, as marginalized populations may be wary of government programs. Although this particular study pertains to Portland, the development of inclusive materials and interventions is a best practice for all cities.

## 5. Conclusions

This study provides compelling evidence that extreme heat exposure is an environmental justice issue. Exposure and adaptive capacity are clearly associated with socio-demographic differences, and many marginalized, low adaptive capacity groups experience disproportionately high temperatures. As low-income populations and non-white populations are presumably most vulnerable to heat-related malady, it is imperative that local governments and practitioners recognize and address social disparities in heat resilience efforts. A detailed local survey process is recommended to overcome limitations of available demographic data, though this study should provide a strong basis for program planning and outreach. Though these results are specific to Portland, Oregon, such relationships likely exist elsewhere, and it is suggested that an environmental justice lens be applied to future study of heat resilience.

## Figures and Tables

**Table 1:** Variables used in covariance analysis. Socio-demographic factors represented as X variables (independent); Heat refuge factors represented as Y variables (dependent).

	Variables
**First Variable (X)**	●% of White
	●% of Black or African American
	●% of American Indian and Alaskan Native (AIAN)
	●% of Asian
	●% of Native Hawaiian and Other Pacific Islander (NHPI)
	●% of Hispanic or Latino
	●% of residents under age 5 (young children)
	●% of residents over age 65 (elderly)
**Second Variable (Y)**	●Urban Heat Index (UHI)
	●Central air-conditioning units (CAC)/km^2^
	●Accessibility to public heat refuges (fast, average, and slow walking speeds)

**Table 2:** Results of Student’s *t*-tests: Statistical significances. Tests which have a *p*-value greater than 0.05 are considered to have failed to reject H_0_.

Variable	Description	*t*-Statistic	*p*-Value	95% Interval—Low	95% Interval—High	Conclusion
**Extreme Poverty**	Percent of population below 50% of the poverty line	2.0848	0.0378	0.009 °C	0.317 °C	Reject H_0_
**High Racial Diversity**	Percent of population who do not identify as ‘white’	5.7579	1.565× 10^−8^	0.274 °C	0.558 °C	Reject H_0_
**Low Education**	Percent of population without a high school diploma or equivalent	7.8371	3.359× 10^−14^	0.402 °C	0.672 °C	Reject H_0_
**Isolated Elderly**	Percent of population who are 65+ years old and live alone	−0.0709	0.994	−0.221 °C	0.206 °C	Fail to Reject H_0_
**Poor English Skills**	Percent of population with poor English speaking abilities	6.0897	2.446× 10^−9^	0.297 °C	0.580 °C	Reject H_0_

**Table 3:** Percentage of the city having access to one or more public heat refuges (cooling centers).

	Slow Walking Speed	Average Walking Speed	Fast Walking Speed
Access to Public Refuges	3.4%	16.9%	32.7%

**Table 4:** Results of covariance analysis: relationship between socio-demographic factors (independent variable), heat exposure, and refuge (dependent variables).

	Socio-Demographic Indicators							
	White	Black	AIAN	Asian	NHPI	Hispanic	Children	Elderly
Expected Temperature Change (C)—for single unit increase of socio-demo indicators	−1.515 **	1.949 **	3.089	1.008	3.471 **	2.010 **	4.620 **	−2.114 **
Central Air Conditioning (CAC units)	96.196 **	−90.034	−552.782	−45.506	−339.324	−111.992	−305.086 *	58.325
Refuge:								
Fast Speed	−1.290 *	5.917 **	6.104	−4.839 **	1.146	−0.767	1.125	−4.103 **
Average Speed	−0.365	2.175 **	2.779	−2.043 **	0.293	−0.492	0.666	−1.764 **
Slow Speed	−0.004	0.293 *	0.280	−0.370 **	−0.056	−0.069	0.144	−0.390 **

Note: * (*p* ≤ 0.05); ** (*p* ≤ 0.01).
